# Comparing different dosing regimens of bevacizumab in the treatment of neovascular macular degeneration: study protocol for a randomised controlled trial

**DOI:** 10.1186/s13063-015-0608-2

**Published:** 2015-03-10

**Authors:** Alexander JE Foss, Margaret Childs, Barnaby C Reeves, Theo Empeslidis, Paul Tesha, Sushma Dhar-Munshi, Samah Mughal, Lucy Culliford, Chris A Rogers, Wei Tan, Alan Montgomery

**Affiliations:** Department of Ophthalmology, Queen’s Medical Centre, Middleton Boulevard, Nottingham, NG7 2UH England; Nottingham Clinical Trials Unit, Queen’s Medical Centre, C-Floor, South Block, Nottingham, NG7 2UH England; Clinical Trials and Evaluation Unit, University of Bristol, Bristol Royal Infirmary, Level 7, Bristol, BS2 8HW England; Leicester Royal Infirmary, Infirmary Square, Leicester, LE1 5WW England; Lincoln County Hospital, Greetwell Road, Lincoln, LN2 5QY England; Department of Ophthalmology, Kings Mill Hospital, Mansfield Road, Sutton-in-Ashfield, NG17 4JL England

**Keywords:** Age-related macular degeneration, Avastin®, Bevacizumab, Trial

## Abstract

**Background:**

Bevacizumab (Avastin®) is as effective as ranibizumab (Lucentis®) in the treatment of neovascular age-related macular degeneration (nAMD). However it has two important structural differences. First, it has two active sites instead of one; second, it retains the Fc portion of the antibody which would be expected to confer a significantly longer half-life. These agents have been associated with systemic complications including strokes, so it is desirable to use the smallest effective dose. Furthermore, the standard dosing regimen requires monthly hospital visits, which present a significant challenge both to the hospital services and to the patients (who are elderly).

**Methods/Design:**

Patients ≥50 years who are eligible for anti-vascular endothelial growth factor (VEGF) treatment of nAMD in the NHS, who are either newly referred for treatment or have reactivation of nAMD and who have not received treatment to either eye for the previous six months.

We have designed a factorial multi-centre masked randomised controlled trial using bevacizumab as the intervention, with patients randomised to one of four arms: to standard or low dose and to monthly or two-monthly patient review. The aim is to recruit sufficient patients (around 1,000) to obtain 304 patients meeting the endpoint over a four-year period. The primary endpoint is time to treatment failure to be analysed using Cox regression.

**Discussion:**

This randomised control trial will show if half dose and two monthly as required is as effective as full dose and monthly regimes. A two monthly as required regimen of Bevacizumab would significantly reduce both the cost and the service delivery burden for the treatment of nAMD while a reduced dose would be expected to enhance the safety profile of this treatment regime.

**Trial registration:**

International Standard Randomised Controlled Trial Number: ISRCTN95654194, registered on 22 September 2009.

## Background

The neovascular form of age-related macular degeneration (nAMD) is a major public health issue, with an estimated 26,000 people being eligible for treatment each year in the United Kingdom [1]. Untreated, this disease has a poor prognosis, with an average visual loss of one to three lines at three months from diagnosis, and three to four lines by one year, as measured by the logMAR visual acuity chart [2]. The introduction of anti-vascular endothelial growth factor (VEGF) agents have revolutionised the treatment for this condition.

Introduction of the pan-VEGF inhibitors, bevacizumab (Avastin®) and ranibizumab (Lucentis®) has transformed the prognosis of nAMD. The first to be developed was bevacizumab, which was an immunoglobulin G (IgG) monoclonal antibody directed against VEGF. It was shown to have powerful anti-angiogenic effects in an experimental tumour model [3] and was developed as an anti-cancer drug. Ranibizumab was derived from the Fab fragment of bevacizumab, which is a fragment of the antibody that has the VEGF binding site.

Two landmark phase 3 trials, Minimally Classic/Occult Trial of the Anti-VEGF Antibody Ranibizumab in the Treatment of Neovascular Age-Related Macular Degeneration (MARINA) and Anti-VEGF Antibody for the Treatment of Predominantly Classic Choroidal Neovascularization in Age-Related Macular Degeneration (ANCHOR), demonstrated that ranibizumab gave large treatment benefits [4,5]. Ranibizumab was administered by monthly intravitreal injection into the vitreous cavity. At 12 and 24 months, more than 90% of eyes treated with ranibizumab (0.5 mg) remained within 15 letters (three lines) of the presenting log MAR visual acuity chart compared to fewer than 64% of eyes treated with photodynamic therapy (ANCHOR) [4], or 62% of eyes treated with sham injections (MARINA) [5]. Of equal importance, eyes treated with ranibizumab showed on average an increase in visual acuity (as measured by the logMAR visual acuity chart) of between five and 10 (one to two lines), and about 35% had logMAR acuity scores better than 70 letters (Snellen equivalent of six out of 12) compared to eyes that had sham or other treatments; this level of vision is compatible with visually demanding tasks such as fluent reading and driving.

Three large randomised control trials, Comparison of Age-related Macular Degeneration Treatment Trial (CATT) [6,7], alternative treatments to Inhibit VEGF in Age-related choroidal Neovascularization IVAN [8,9] and Groupe d'Etude Français Avastin versus Lucentis dans la DMLA néovasculaire (GEFAL) [10], have demonstrated non-inferiority of bevacizumab compared to ranibizumab with respect to a non-inferiority margin of five letters.

There are two structural differences (see below) between bevacizumab and ranibizumab, and these differences provide the rationale for the “Randomised controlled trial of high and low dose Avastin® for Neovascular Macular Degeneration in the East Midlands” (TANDEM) trial, which uses a two-by-two factorial design to investigate dosing and review and treatment frequency. This is explained in the next two sections.

### Rationale for two-monthly arms and the issues of health care delivery and ocular safety

The first factor compares monthly versus bimonthly review. This factor is based on the observation that bevacizumab retains the IgG Fc region. Molecules that possess the Fc region have a long half-life *in vivo* [11-13]. The existing data support the hypothesis that bevacizumab has a longer-half life than ranibizumab. The half-life of ranibizumab in the vitreous of rabbits is 2.9 days [14,15] compared to a half-life of 4.3 days for bevacizumab [14]. In humans, the reported half-life of bevacizumab is 10 days [16] compared to the reported half-life for ranibizumab of three days in primate eyes [16]. The ABC trial showed that patients monitored and treated with bevacizumab on a six-weekly basis had similar results to the ANCHOR and MARINA trials [17]. In this context, it also is of interest that aflibercept, which also has the structural motif of retaining the IgG Fc portion, has been shown to be effective when given bimonthly [18,19].

Similar results to those seen in the ANCHOR and MARINA trials can be achieved without continuous dosing. The Prospective Optical Coherence Tomography (OCT) Imaging of Patients with Neovascular Age-Related Macular Degeneration (AMD) Treated with intraOcular Ranibizumab (PrONTO) trial showed that a reduction in treatment frequency can be achieved through rigorous tailoring of treatment to morphological parameters, with comparable visual acuity outcomes [20,21].

Bimonthly dosing has not been investigated for ranibizumab. The phase IIIb study of ranibizumab efficacy and safety in choroidal neovascularization due to age-related macular degeneration (PIERtrial showed that assessment and re-treatment with ranibizumab once every three months still gave good results, but not equal to those achieved with monthly review [22]. The mean duration of clinical action of bevacizumab has been reported to be significantly longer than ranibizumab at 100 days [23], and this suggests that monthly assessment and potential treatment may be too frequent, and that a review frequency of around every 56 days (eight weeks) should be sufficient. Two-monthly dosing has the additional advantage of less frequent exposure to the risk of local complications related to the injection process.

### Rationale for low dose arms and the issue of systemic safety

There is a second structural difference between bevacizumab and ranibizumab. Bevacizumab has two binding sites to VEGF whereas ranibizumab, being the Fab fragment, has only one; however, it should also be noted that the target ligand is also bivalent. Binding studies of the whole molecules of bevacizumab and ranibizumab are broadly similar [24]. It is not clear how an ‘equivalent’ dose should be calculated given that the target ligand is also bivalent.

The most commonly used dose of bevacizumab, 1.25 mg, was calculated on the assumption that the dose should be equimolar with respect to ranibizumab; this calculation did not take into account the fact that bevacizumab has two, rather than one, binding sites. Increasing the dose of bevacizumab offers no increased clinical benefit; doses of both 1.25 mg and 2.5 mg are equally effective in the treatment of AMD [25,26]. Equally, increasing the dose of ranibizumab to 2.0 mg from the standard dose of 0.5 mg did not result in a superior clinical result [27,28]. Moreover, there is evidence that a lower dose may be just as effective. In the context of diabetes, Avery *et al*. have shown that proliferative disease will respond to an injected dose as low of 0.00625 mg (6.25 μg) [29].

Concerns that these agents may cause both systemic [7] and local complications, such as geographic atrophy [30], make it desirable to use the smallest clinically effective dose. The combination of a half-dose administered two-monthly would result in a potential four-fold lowering of the total drug delivered during the maintenance phase. The evidence described above suggests that this is still a large enough dose to give a good clinical response.

## Methods/Design

### Study design

The trial is designed to test two hypotheses:

1. Low dose bevacizumab is not inferior to standard dose bevacizumab with respect to maintenance of visual acuity.
2. Following an initial three-month period of monthly review, eyes reviewed every two months will not be inferior to those reviewed monthly, with respect to maintenance of visual acuity.

**Figure 1:**
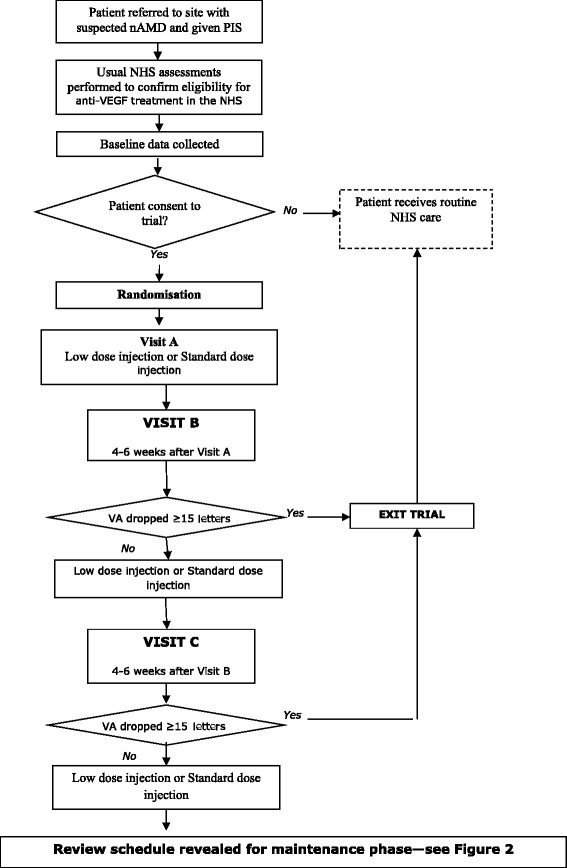
**Flow diagram for the induction phase of the trial protocol.** nAMD: neovascular Age Related Macular Degeneration. PIS: patient information sheet. VA: visual acuity.

These hypotheses will be investigated using a two-by-two factorial design. On entry into the trial, all patients will be randomised between standard dose (1.25 mg) or half dose (0.625 mg) of bevacizumab, and review every four to six weeks or every eight to 10 weeks. All patients will initially undergo three-monthly injections, and during this phase the allocated frequency of subsequent review will be masked from doctor and patient. This phase is called the induction phase and is summarised in Figure 1.

On completion of the induction phase the review frequency will be revealed and the patients will then enter the maintenance phase. During this phase, re-treatment is determined on the basis of optical coherence tomography (OCT) findings, but treatment failure is assessed on the basis of the logMAR visual acuity score. In both arms, the treatment failure criteria are assessed every eight to 10 weeks to avoid introducing bias by reviewing one group more frequently.

The trial is designed to reflect current clinical practice. There are no fixed criteria for patient discharge and each participating unit can follow their own discharge policy. Patients who are discharged from clinic are considered to be ‘hibernating’, and if the disease reactivates then they will be allowed to re-enter the trial, keeping their original randomisation. These re-entering patients repeat the induction phase and a new baseline is established on re-entry.

**Figure 2:**
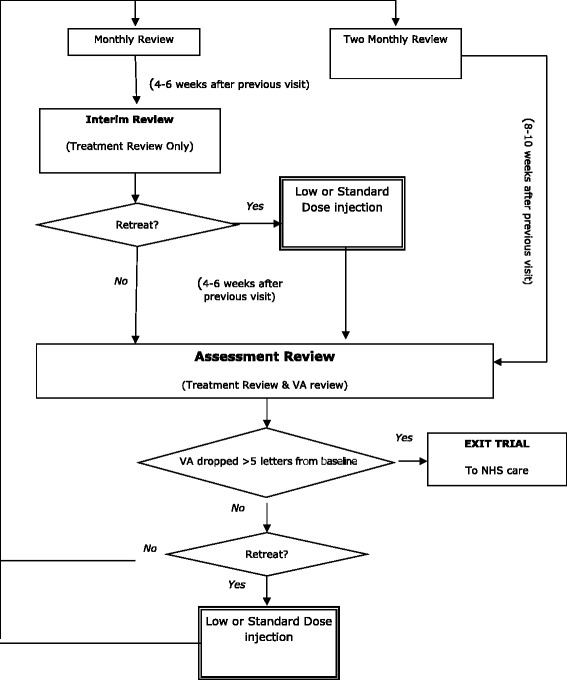
**Flow diagram showing the maintenance phase of the trial protocol.** VA: visual acuity.

Similarly, patients who develop bilateral disease remain in the trial. The fellow eye is treated according to the same allocation as the first eye and undergoes the initial three-month period of monthly review followed by maintenance. The maintenance phase is summarised in Figure 2.

### Inclusion and exclusion criteria

This trial was designed to be as inclusive as possible, thereby representing the patient population that presents to the NHS requiring treatment for nAMD. It is a multi-centre trial, with a number of participating hospitals located in the East Midlands including Chesterfield, Derby, Leicester, Lincoln, Mansfield and Nottingham.

The inclusion criteria reflect National Institute for Health and Care Excellence (NICE) guidelines for treatment of nAMD [31], and are:

1. Age ≥50 years,
2. Newly referred for treatment of nAMD or reactivation of nAMD,
3. No treatment for nAMD to either eye for the previous six months and
4. Eligible for anti-VEGF treatment of nAMD in the NHS.

The exclusion criteria are those that apply to all patients being considered for treatment with an anti-VEGF agent, namely:

1. Known hypersensitivity to recombinant human or humanised antibodies,
2. Woman of child bearing potential and not willing to use contraception,
3. Male with spouse of child bearing potential not willing to use condoms or
4. Pregnant or breast feeding.

The NICE guidelines state that patients with nAMD are eligible for treatment with vision between six out of 12 and six out of 96 [31]. It is not standard practice in the NHS for all patients to have a refraction before checking their vision, however it is for entry into the TANDEM trial. Accordingly, patients approached about the trial who meet the NICE guidelines on the basis of vision testing with their current glasses (and would have been treated in the normal NHS environment) remain eligible for the trial even if their vision improves to better than 6/12 with the refraction that occurs as part of the trial protocol. This decision was based on two considerations. First, it was considered ethically difficult to withdraw the offer of treatment once made, and second, consideration was for the trial population to as accurately reflect the patient population being treated under the NHS as was reasonable.

### Trial participation

In view of the age and study population it will be expected that the treating clinicians consider and determine whether the potential trial patients have the capacity to give fully informed consent to enter into the trial. If the patient fulfils the eligibility criteria and agrees to enter the trial, written informed consent is obtained. All patients will receive trial participation information at least 24 hours before being asked to give informed consent. Patients who do not wish to consent to the trial will be treated in the NHS according to standard care.

### Randomisation and masking

Randomisation is stratified by centre and blocked using random permuted blocks of varying size. Allocation sequences are computer generated, and concealed from staff recruiting participants to the trial using a secure internet-based system created and maintained by Nottingham Clinical Trials Unit. Information to identify a participant uniquely and to confirm eligibility must be entered before the system assigns a study number and treatment allocation.

Participants and clinic staff providing care (ophthalmologists, optometrists and nurses) are masked to the bevacizumab dose. Patient allocation to monthly or two-monthly review intervals is masked until three months following allocation, but not thereafter.

### Source of trial drug

The bevacizumab syringes are manufactured, prepared and shipped by The Royal Liverpool and Broadgreen University Hospital via individually boxed syringes with a tear-off sticker indicating the bevacizumab dose. Following preparation of the prescription, the identifying label is removed by the pharmacist, masking the syringe prior to dispensing. The unique patient identifier is recorded on the remaining masked label to ensure the correct patient receives their allocated randomised treatment via their intravitreal injection.

### Data collection

Data collection comprises the following activities:

1. Baseline data collection (including general and ophthalmic history, examination and baseline morphology of nAMD lesion) at the initial visit;
2. Refraction at the initial visit and only at subsequent visits, if required;
3. Early Treatment in Diabetic Retinopathy Treatment Study (ETDRS) logMAR visual acuity score, including checking visual acuity against criteria for vision deterioration at the first three visits and at the assessment reviews, but not at interim reviews unless clinically indicated. Patients whose vision dropped to the point that they meet the outcome measure will have their visual acuity checked by an independent observer;
4. Fundus fluorescein angiogram (FFA) and colour photography is performed at the initial visit (images sent for independent grading); then at each subsequent visit if required (but not sent for grading);
5. OCT and ocular examination is performed at the initial visit and at assessment and interim reviews, along with assessment for need for re-treatment (all visits after visit C) and
6. Assessment of (serious) adverse events at all visits.

Separate screening and enrolment logs are maintained for all patients screened and/or enrolled into the trial in all participating centres. After a visit has taken place, centre staff transfer data into a secure web-based database within five working days. Baseline FFA and colour images are submitted for grading.

### Image grading

A baseline colour fundus photograph and fluorescein angiogram will be exported to the Network of Ophthalmic Reading Centres (NetwORC) UK for image grading. The angiographic grading will provide the area of active neovascularisation (classic and any late leakage that occurs at the site of irregular elevation of the retinal pigment epithelium which will be considered as arising out of an occult membrane located between the retinal pigment epithelium (RPE) and the choroid). The area of blocked fluorescence at the margins of the leakage which could represent occult choroidal neovascularisation (CNV) will be measured separately. The area of blocked fluorescence will be matched with the colour image to ensure that if this is due to blood, this area will be outlined and reported separately to any blocked fluorescence not due to blood. If areas of hyper fluorescence are noted on the FFA adjacent to the optic disk and associated with focal elevations of the RPE, the grading form will include a comment indicating that there is a high degree of suspicion that the lesion could represent polypoidal choroidopathy. Similarly if reticular drusen, choroidal thinning and intraretinal blood are observed and the fluorescein leakage is dense and rapid, this will raise a high index of suspicion of a retinal angiomatous proliferation (RAP) lesion.

### Outcome

The primary outcome is time to treatment failure, defined as loss of more than five letters (logMAR visual acuity chart) from the baseline established as the average of the visual acuities at the first three visits. The primary analysis will be at the margins, unless there is evidence of an interaction, in which case low dose plus bimonthly, low dose plus monthly and standard dose plus bimonthly will each be compared with standard dose plus monthly. There will be no correction for multiple comparisons [32].

### Ethics approval

Ethical approval for this trial was granted by the Leicestershire, Northamptonshire and Rutland Research Ethics Committee (NRES reference 09/H0406/86).

### Statistical considerations

#### Sample size

The target sample size is based on the primary hypotheses, with the primary outcome analysed as a time-to-event variable. When designing the study, we assumed that there would be one study eye per participant, because this assumption is conservative and simplifies estimation of sample size. Additionally, we assumed that the annual risk of vision deterioration is 10% if allocated to receive standard dose bevacizumab [9], and 10% if allocated to monthly review intervals, and that there is no interaction between bevacizumab dose and review interval. With a non-inferiority hazard ratio margin of 1.4 for between-arm main effects, 90% power and one-sided 5% alpha, a total of 304 events are required to be observed, and the target sample size for recruitment was 2,000 participants. In January 2014 after three years of recruitment, we reviewed the assumptions underlying the original recruitment target. Based on 437 randomised participants and 374 person-years of observation, we revised the annual event rate of the primary outcome from 10 to 20%, and additionally accounted for annual censoring (death, suspension of treatment following six months of stable disease, withdrawal of consent for study participation or no response to attempted contact) of 16%, which had not been incorporated into the original calculation. The target number of 304 events remains unchanged, but the target number of randomised participants required to achieve this has been revised to around 900 to 1,000.

### Data analysis

Demographic and clinical characteristics at baseline of participants in each of the four factorial cells will be described using appropriate descriptive statistics. The main approach to between-group comparisons will be to analyse all participants as randomised regardless of adherence with allocation. In addition for the primary outcome, a per protocol analysis will be conducted that excludes participants with protocol violations (specifically, failure to collect outcome data or patients who received treatment in addition to the trial intervention, such as ranibizumab). All regression models will include centre as a fixed effect, since this was used in stratifying randomisation.

Since participants may have one or both eyes included in the study, analysis of the primary outcome will be carried out using shared-frailty Cox regression models. Efficiency of the two-by-two factorial design is realised when all participants are included in estimating the effects of both factorial elements. Therefore the analysis will first explore whether the effect of low versus standard dose bevacizumab, and two-monthly versus monthly review interval, differs according to the presence or absence of the other. If there is no evidence of an interaction, main effects will be estimated by including a separate term for each in the model. If there is evidence of an interaction, then the effects of reduced dose alone and increased interval alone will be estimated using the factorial cells, with accordingly reduced sample sizes for these comparisons. Secondary analyses of the primary outcome will include additional adjustment for any variables exhibiting marked imbalance at baseline, and investigation of subgroup effects according to: baseline visual acuity in study eye ≤44 versus >44; 2) baseline CNV size ≤4 versus >4 and nAMD lesion composition. These analyses will be conducted by fitting interaction terms to the regression models. It is recognised that the study is not powered to detect differential treatment effects among subgroups, and these analyses will be viewed as exploratory.

Secondary outcomes will be analysed using a similar approach as for the primary analysis, with regression model appropriate for type of outcome. All between-group comparisons will be described using appropriate estimates of effects (that is, hazard ratio, odds ratio or difference in means, depending on outcome type) and 95% confidence intervals.

## Discussion

The use of the anti-VEGF agents has transformed the prognosis of patients with nAMD. However there are two major barriers to the delivery of health care to this group of patients. The first is the cost and the second is the requirement to be seen monthly. The TANDEM trial is designed to investigate both of these barriers.

The CATT, IVAN and GEFAL trials have shown that bevacizumab is not inferior to ranibizumab in the treatment of nAMD [7,9,10], and there is a strong health economic incentive for its use, notwithstanding the current regulatory situation. However, there are very limited trial data guiding the most beneficial way to use bevacizumab.

The optimum dose for bevacizumab is not known and there is considerable evidence suggesting that a lower dose may be equally beneficial. This is important while concerns over the safety of the pan-VEGF inhibitors remain, as one would expect the lower dose to carry a better safety profile. However, adverse event rates are low and this study is not powered to detect any difference in adverse and serious adverse events.

The second issue is that of frequency. Ophthalmic departments are struggling to meet the one-monthly review schedule that the use of ranibizumab requires. There is reason to believe that it would be effective when given on a two-monthly regimen, which in turn would halve the required number of out-patient assessment visits.

### Trial status

Recruitment to the TANDEM trial is ongoing. First patient was randomised in Novemeber 2010 and recruitment is expected to end at the end of 2016.

## Abbreviations

AMD: Age-related Macular Degeneration
CNV: Choroidal Neovascularisation
FFA: Fundus Fluorescein Angiography
nAMD: Neovascular Age-related Macular Degeneration
NICE: National Institute for Health and Care Excellence
NHS: National Health Service
OCT: Optical Coherence Tomography
RAP: Retinal Angiomatous Proliferation
RPE: Retinal Pigment Epithelium
VEGF: Vascular Endothelial Growth Factor
